# The Effects of Th17 Cytokines on the Inflammatory Mediator Production and Barrier Function of ARPE-19 Cells

**DOI:** 10.1371/journal.pone.0018139

**Published:** 2011-03-30

**Authors:** Ying Chen, Peizeng Yang, Fuzhen Li, Aize Kijlstra

**Affiliations:** 1 Chongqing Key Laboratory of Ophthalmology, Chongqing Eye Institute, The First Affiliated Hospital of Chongqing Medical University, Chongqing, People's Republic of China; 2 Zhongshan Ophthalmic Center, Sun Yat-sen University, Guangzhou, People's Republic of China; 3 Department of Ophthalmology, Eye Research Institute Maastricht, University Hospital Maastricht, Maastricht, The Netherlands; University of Leuven, Rega Institute, Belgium

## Abstract

Th17 cells have emerged as a key factor in the pathogenesis of uveitis as well as other autoimmune disorders. They secrete a number of cytokines including IL-17A, IL-17F and IL-22 and until now the effects of these cytokines on resident cells of the eye were not yet clear. The purpose of this study was to investigate the effects of Interleukin (IL)-17A, IL-17F and IL-22 on the production of inflammatory mediators and barrier function of retinal pigment epithelium cells. We showed that ARPE-19 cells, a spontaneously arisen cell line of retinal pigment epithelium (RPE), constitutively expressed IL-17RC and IL-22R, but not IL-17RA. IL-17A significantly enhanced the production of CXCL8, CCL2, CCL20 and IL-6 by these cells. IL-17F had a similar effect on the production of CXCL8, CCL2 and IL-6 by ARPE-19 cells, but did not influence the expression of CCL20. Both IL-17A and IL-17F significantly decreased the transepithelial electrical resistance (TER) of the ARPE-19 monolayer and increased the diffusion rate of fluorescein isothiocyanate (FITC)-dextran. They also disrupted the distribution of the junction proteins zonula occludens (ZO)-1 and occludin at the interface of adjacent cells. IL-22 did not have a detectable effect on the production of the tested inflammatory mediators by ARPE-19 cells, TER of the ARPE-19 monolayer, the diffusion rate of FITC-dextran or the distribution of ZO-1 and occludin. This study demonstrates that IL-17A and IL-17F, but not IL-22, significantly promoted ARPE-19 cells to secrete inflammatory mediators and compromised the ARPE-19 monolayer barrier function in association with a disrupted distribution of ZO-1 and occludin. These results suggest that both IL-17A and IL-17F may play a role in posterior segment inflammation of the eye.

## Introduction

Th17 cells have been identified as a subset of T helper lymphocytes characterized by the production of a number of cytokines including Interleukin (IL)-17A, IL-17F and IL-22[1], [2]. They have emerged as a key factor in the pathogenesis of uveitis as well as other autoimmune disorders[3], [4]. Growing evidence suggests that Th17 cells trigger inflammatory responses primarily via IL-17A[5], although IL-17F and IL-22 have also been shown to be involved in the pathogenesis of autoimmune diseases[6], [7], [8], [9]. It has been well established that these cytokines exert their role through an interaction with their receptors in certain cells. IL-17RA is expressed ubiquitously with a higher level in hemopoietic cells, whereas IL-17RC is mainly expressed by nonhemopoietic cells in tissues such as adrenal gland, prostate, liver and thyroid[10]. IL-22R is primarily expressed in epithelial cells [11].

Recent studies have shown that IL-17A is involved in the development of inflammation through promoting the production of chemokines and proinflammatory cytokines including CXCL8, CCL2 and IL-6[12]. IL-17F has biological activities similar to IL-17A[13]. IL-22 has a weaker ability to induce the expression of CXCL8, CCL2 and CCL20 by keratinocytes or endothelial cells[8], [14], [15].

A recent study showed an increased expression of IL-17A mRNA in the retina of mice with experimental autoimmune uveoretinitis (EAU), a classical model for human autoimmune uveitis[3]. IL-17A protein and IL-22 mRNA were found to be highly expressed by peripheral blood mononuclear cells (PBMCs) from uveitis patients[7], [16], [17]. However it is not yet known which cells are the targets of these cytokines and how they function within the eye during uveitis involving the posterior segment. It has been well documented that the retinal pigment epithelium (RPE) plays an important role in the maintenance of the ocular immunomicroenviroment as well as in the pathogenesis of uveitis[18]. It has been shown that RPE could secrete a number of chemokines and proinflammatory cytokines including CXCL8, CCL2 and IL-6 in response to various stimulants[18]. These inflammatory mediators produced by RPE are generally similar to those produced via IL-17A and IL-17F stimulation[12], [13], [19]. This similarity stimulated us to investigate the effects of these cytokines on ARPE-19, a spontaneously arisen cell line of RPE, which has been extensively used in the past decades to investigate the role of this cell layer in the pathogenesis of a number of diseases including age related macular degeneration, vitreoretinopathies and uveitis. Our study revealed that ARPE-19 cells constitutively expressed IL-17RC and IL-22R. IL-17A was able to stimulate the production of CXCL8, CCL2, CCL20 and IL-6 by ARPE-19 cells. IL-17F only stimulated the secretion of CXCL8, CCL2 and IL-6 by these cells. Both IL-17A and IL-17F were able to compromise the barrier function of the ARPE-19 monolayer in association with a disrupted distribution of the tight junction proteins zonula occludens (ZO)-1 and occludin.

## Results

### Expression of IL-22R, IL-17RC and IL-17RA by ARPE-19 Cells

**Figure 1:**
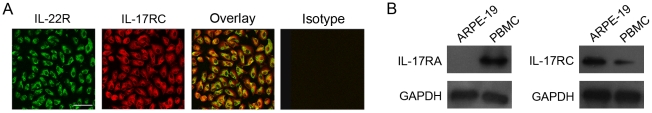
Expression of IL-22R and IL-17RC in ARPE-19 cells. (A) Coexpression of IL-22R and IL-17RC in ARPE-19 cells was shown by immunocytochemistry. Scale bar  = 75 µm. (B) Western blot revealed that ARPE-19 cells expressed only IL-17RC. However, PBMCs expressed both IL-17RA and IL-17RC.

Immunocytochemistry was used to detect IL-17RA, IL-17RC and IL-22R expression by ARPE-19 cells. The results showed that all cells expressed IL-17RC and IL-22R. The expression of both IL-17RC and IL-22R was uniquely distributed on the cellular membrane and in the cytoplasm but not in the nucleus(Fig. 1A). IL-17RA expression was not detectable. There was no staining in the isotype control experiments. Additional experiments were performed to verify the absence of IL-17RA using Western blot analysis and including PBMCs as a positive control. The results confirmed our immunocytochemistry findings and showed that ARPE-19 cells expressed only IL-17RC but not IL-17RA(Fig. 1B). However, PBMCs expressed both IL-17RA and IL-17RC.

### The Influence of IL-17A, IL-17F and IL-22 on the Production of Inflammatory Mediators by ARPE-19 Cells

**Figure 2:**
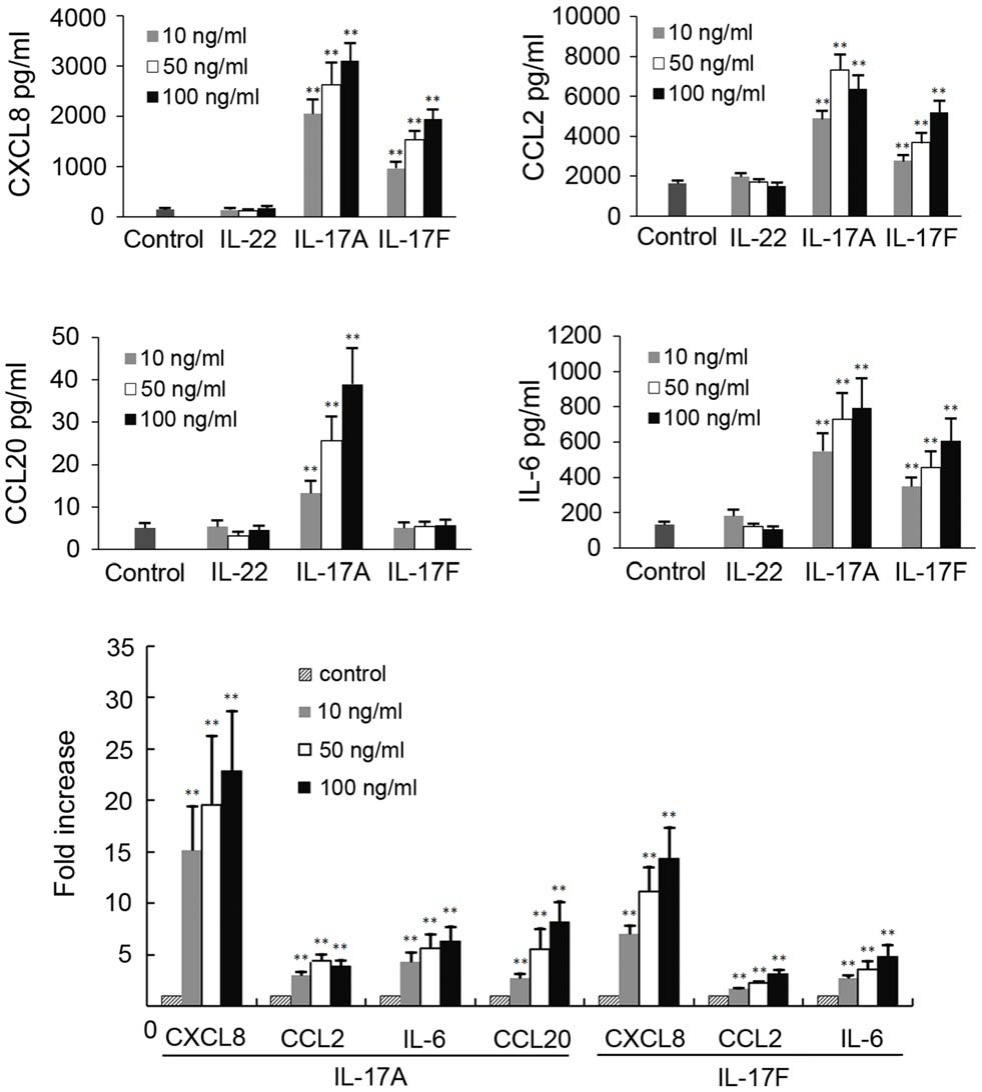
IL-17A and IL-17F but not IL-22 promoted chemokines and IL-6 production in ARPE-19 cells. Confluent ARPE-19 cells were stimulated with different concentrations of IL-17A, IL-17F or IL-22 as indicated. After 24 hours of incubation, protein concentrations of CXCL8, CCL2, CCL20 and IL-6 released into the supernatants were measured by ELISA. Data were shown as the means±SEM of four independent experiments. **p<0.01 versus the control group.

To determine the influence of IL-17A, IL-17F and IL-22 on the production of inflammatory mediators by ARPE-19 cells, the expression of CXCL8, CCL2, CCL20, CXCL10 and IL-6 by these cells was assayed. The results showed that IL-17A significantly enhanced the production of CXCL8 (p<0.001), CCL2 (p≤0.001), CCL20 (p<0.001) and IL-6 (p<0.001), but did not influence the production of CXCL10 (data not shown) by these cells (Fig. 2). The stimulation of IL-17A on these mediators appeared in a dose-dependent manner. IL-17F had a similar, but somewhat weaker, stimulatory effect on the production of CXCL8, CCL2 and IL-6 in a dose-dependent manner. However it did not have any influence on the production of CCL20 and CXCL10. CXCL8 production induced by both IL-17A and IL-17F was the most prominent, followed by IL-6 and CCL2. There was no effect of IL-22 on the production of all the investigated inflammatory mediators at the tested three concentrations.

### The Influence of IL-17A, IL-17F and IL-22 on the Barrier Integrity of the ARPE-19 Monolayer

**Figure 3:**
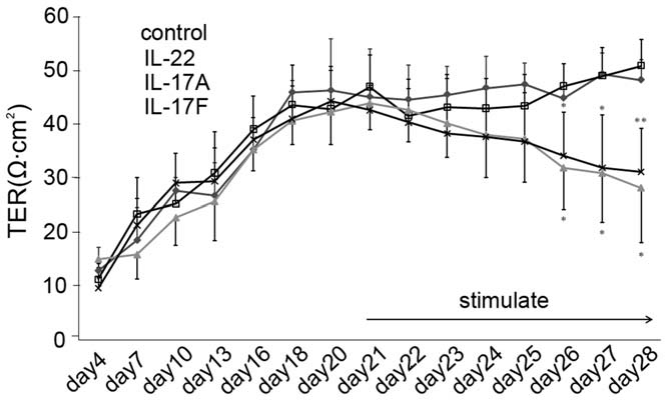
Effect of IL-17A, IL-17F or IL-22 on TER of cultured ARPE-19 monolayer. Monolayers were cultured for 21 days, where after the various stimuli were added. Incubation of ARPE-19 monolayers with 50 ng/ml IL-17A or IL-17F induced a gradual decrease of TER, and a significant effect occurred 5 days (p = 0.019, p = 0.045) after stimulation. The continuous decreases were also observed 6 days (p = 0.01, p = 0.016) and 7 days (p = 0.023, p = 0.008) after stimulation. IL-22 had no effect on TER. Data were shown as the means±SEM of four independent experiments. *p<0.05 versus the control group.

**Figure 4:**
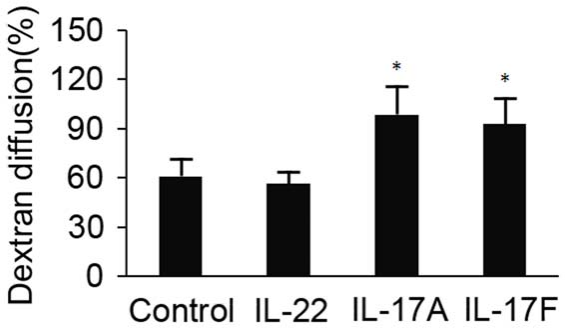
Effect of IL-17A, IL-17F or IL-22 on transepithelial diffusion rate of FITC-dextran in ARPE-19 monolayer. Stimulation of ARPE-19 monolayer with 50 ng/ml IL-17A, IL-17F or IL-22 for 6 days induced a higher FITC-dextran diffusion rate at 24 hours compared with the control group. A diffusion percentage approaching 100% indicates that the amount of dextran-FITC in the upper and lower chamber approaches the same values. IL-22 had no effect on diffusion rate. Data were shown as the means±SEM of four independent experiments. *p<0.05 versus the control group.

The transepithelial electrical resistance (TER) and permeability of the ARPE-19 monolayer was used to evaluate the influence of IL-17A, IL-17F or IL-22 on the barrier integrity of the monolayer. The result revealed that the TER of the ARPE-19 monolayer grown on transwell filters increased rapidly during the initial 18 days of culture and reached a plateau within the following 3 days (Fig. 3). A mean level of 45.0±6.0 Ω/cm^2^ TER was recorded on day 21. On day 21 the monolayer was stimulated with IL-17A, IL-17F and IL-22 respectively for 7 days and the TER of the ARPE-19 monolayer was measured every day during this time period. It was found that IL-17A or IL-17F gradually decreased the TER, and a significantly decreased TER occurred on day 5 (p = 0.019, p = 0.045 respectively) and thereafter. The monolayer permeability was further assayed by measuring the transepithelial diffusion rate of fluorescein isothiocyanate (FITC)-dextran through the ARPE-19 monolayer grown on the filters. A significantly increased diffusion rate of FITC-dextran was observed when the cells were cultured in the presence of IL-17A (p = 0.01) or IL-17F (p = 0.015) (Fig. 4). We failed to find an influence of IL-22 on the TER and permeability of the ARPE-19 monolayer.

### The Influence of IL-17A, IL-17F and IL-22 on the Expression of ZO-1 and Occludin

**Figure 5:**
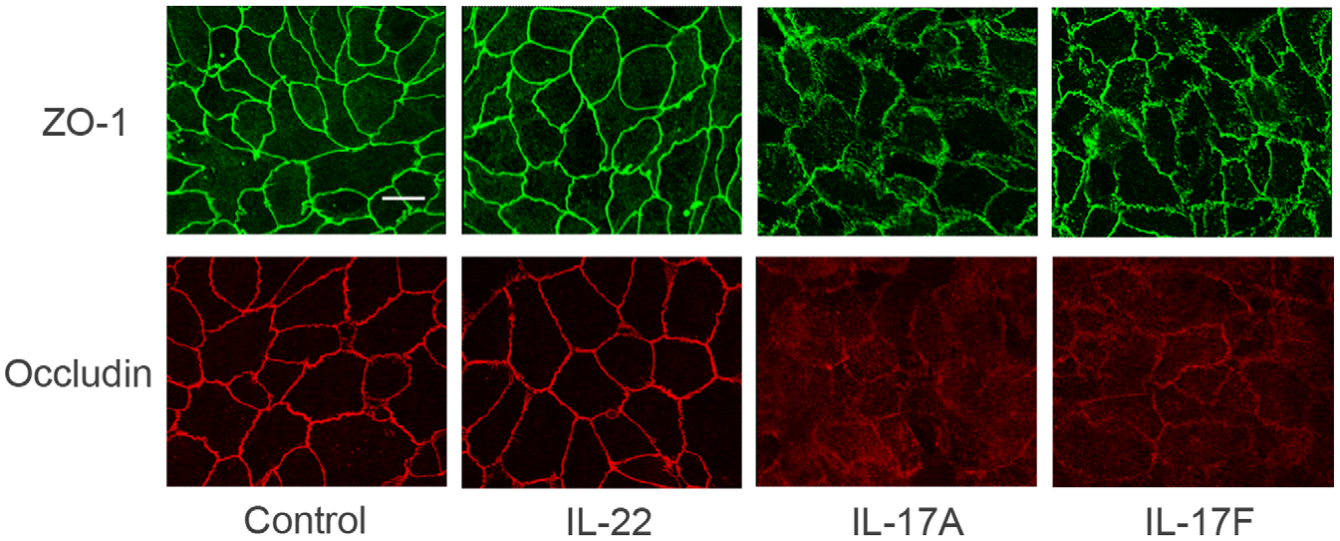
Effect of IL-17A, IL-17F or IL-22 on the distribution of junction proteins in ARPE-19 monolayer. Cells were incubated with or without 50 ng/ml IL-17A, IL-17F or IL-22 for 6 days, then fixed and immunolabeled with ZO-1 or occludin. Immunostaining for ZO-1 and occludin in untreated or IL-22-treated ARPE-19 monolayer showed a continuous labeling in the region of cell-cell contact. Incubation with IL-17A or IL-17F caused a marked disruption of ZO-1 and occludin staining. The immunostaining shown is representative of three independent experiments. Scale bar  = 15 µm.

As the aforementioned experiment showed a compromised barrier function following exposure to IL-17A and IL-17F, a further study was performed to examine whether this abnormality was associated with a disturbed distribution of ZO-1 and occludin, two proteins critical to the barrier integrity. The results showed that the expression of ZO-1 and occludin was continuous and regular around the cells in the ARPE-19 monolayer cultured alone. The exposure of these cells to IL-17A or IL-17F for 6 days resulted in a markedly disturbed distribution of ZO-1 and occludin. The abnormal distribution of ZO-1 typically manifested as fragmental staining. The staining of occludin showed a diffuse cytoplasmic distribution (Fig. 5). 4′,6-diamidino-2-phenylindole (DAPI) staining for nuclei showed the uniform distribution of ARPE-19 cells over the monolayer. Exposure to IL-22 was similar to that in the ARPE-19 monolayer cultured without any stimuli.

## Discussion

This study shows that ARPE-19 cells constitutively express IL-17RC and IL-22R at the protein level. IL-17A and IL-17F, but not IL-22, significantly stimulated ARPE-19 cells to secrete a number of inflammatory mediators such as CXCL8, CCL2, CCL20 and IL-6. Additionally IL-17A and IL-17F significantly compromised the barrier function of the ARPE-19 monolayer and disturbed the distribution of the tight junction proteins ZO-1 and occludin in the ARPE-19 monolayer. Posterior segment inflammation of the eye is accompanied by T cell infiltration and recently the Th17 subpopulation has been implicated to play an important role in the pathogenesis of this blinding condition[3], [4], [20]. Th17 cells secrete a number of cytokines including IL-17A, IL-17F and IL-22[2] and until now the effects of these cytokines on resident cells of the eye was not yet clear and was therefore the subject of our study.

We showed that the ARPE-19 cells expressed IL-17RC and IL-22R, but not IL-17RA, although IL-17RA is generally thought to be essential for IL-17A function[21]. A recent study however showed that IL-17RC was able to bind to both IL-17A and IL-17F with a similar high affinity[10]. Our results were consistent with earlier studies by Leung et al.[22] who found that ARPE-19 cells and RPE cells from primary cultures (2nd–3rd passage) only expressed IL-17RC mRNA, but definitely not IL-17RA mRNA. Mouse RPE cells, on the other hand, do express IL-17RA[23]. This discrepancy could be explained by the origin of the RPE cells used in the experiments. These findings suggest that ARPE-19 cells are one of the targets for IL-17A, IL-17F and IL-22. Both IL-17A and IL-17F were able to profoundly stimulate the production of CXCL8, CCL2 and IL-6 by ARPE-19 cells, albeit that on a quantitative basis IL-17F was shown to be less active. CXCL-8 and CCL2 have been demonstrated as important chemokines for neutrophils and monocytes respectively. IL-6 also has a variety of proinflammatory biological activities and has been shown to play an important role in the pathogenesis of inflammation[24]. This result suggests that both IL-17A and IL-17F may be involved in posterior segment inflammation of the eye through stimulation of inflammatory cytokine production by the resident cell population. It was interesting to note that IL-17A, but not IL-17F, upregulated the expression of CCL20, a chemokine involved in the attraction of Th17 cells[25], [26]. The inconsistent effects of IL-17A and IL-17F were also observed by Zrioual et al. in human rheumatoid arthritis synoviocytes[27]. They found that although both IL-17A and IL-17F had similar chemokine inducing properties, the activity of IL-17F was less powerful. It is worthwhile to point out that IL-22 did not have a detectable effect on the production of all the investigated cytokines at the three tested concentrations although its receptor was expressed by these cells. A recent study showed an increased expression of IL-22 by PBMCs from uveitis patients, suggesting its possible involvement in this disease[7]. It is not clear whether IL-22 could influence the production of these aforementioned cytokines under other culture conditions or the production of other cytokines by these ARPE-19 cells. More studies are needed to clarify these issues.

As the integrity of the RPE barrier is critical for the interphase between the retina and choroid, a further study was designed to investigate whether IL-17A, IL-17F and IL-22 could break down this barrier. TER has been widely used to evaluate the barrier function of epithelium in vitro[28], [29], [30]. In the present study, TER of the ARPE-19 monolayer gradually increased and reached a plateau after 3 weeks of culture. Addition of IL-17A or IL-17F resulted in a decrease of the TER of the ARPE-19 monolayer. This result suggests that both cytokines could significantly compromise the barrier function of the ARPE-19 monolayer. Transepithelial fluxes of FITC-dextran, a test to evaluate the structural integrity of cultured epithelium monolayers[30], [31], was also used in the present study. Exposure of IL-17A or IL-17F for 6 days resulted in a significantly increased diffusion rate as compared to unstimulated controls. This result suggests that both IL-17A and IL-17F had an effect on the structural integrity of the monolayer. These alterations on TER and diffusion rate of FITC-dextran were also reported previously when RPE were exposed to IL-1β or to tumor necrosis factor-α combined with lipopolysaccharide and interferon-γ[28], [29]. Interestingly we did not find any effect of IL-22 on both TER or the diffusion rate of FITC-dextran. This result, together with that observed in the cytokine study, suggests that ARPE-19 cells may not be an important target of IL-22 with respect to the production of the tested cytokines and the influence on barrier function under the culture conditions described in the present study. It is worthwhile to point out that a decreased TER was reported in an experiment using human fetal RPE cells in the presence of IL-22.[7]. Additionally, induction of RPE cell apoptosis by IL-22 was also observed in this study. The difference with regard to the TER results between both studies may be, at least partially, explained by the different origin of the target cells or rIL-22 used.

Barrier function of epithelium is brought about by intercellular tight junctions, involving a number of tight junction proteins. ZO-1 and occludin are considered as two important tight junction proteins of the RPE cell layer and their expression was used to correlate whether the breakdown of ARPE-19 monolayer barrier function as found in this study was associated with an abnormal distribution of both proteins. Correspondingly we found that both IL-17A and IL-17F were able to disturb the distribution of these two proteins. The abnormal distribution of both proteins caused by IL-17A or IL-17F, although not completely similar, may lead to a decreased TER and increased diffusion rate of FITC-dextran. Similar to the results observed in the studies of cytokines, TER and diffusion rate of FITC-dextran, IL-22 did not affect the distribution of the tested two tight junction proteins.

In conclusion, the present study revealed the presence of IL-17RC and IL-22R on cultured ARPE-19 cells. IL-17A and IL-17F significantly increased the expression of a number of inflammatory mediators by these cells. Both cytokines profoundly disturbed the distribution of ZO-1 and occludin and consequently affected the barrier function of the ARPE-19 monolayer. IL-22 influenced neither the production of the tested cytokines, nor the barrier function of the ARPE-19 cells. The exact mechanisms whereby IL-17A and IL-17F affect the function of RPE cells needs to be addressed in future studies. As the biological function of the ARPE-19 cell line may be different from RPE in vivo, the in vitro data shown in the study presented here need to be confirmed using in vivo models

## Materials and Methods

### Cell Culture

Human ARPE-19 cells were obtained from the American type culture collection (ATCC), and cells between passages 16 and 20 were used for experiments. The cells were cultured in 1∶1 in Dulbecco modified Eagle medium/F12(DMEM/F12;1∶1, Invitrogen, Beijing, China) with 10% fetal bovine serum (FBS, Invitrogen, Carlsbad, CA), 100 U/mL penicillin, and 100ìg/ml streptomycin in a humidified incubator at 37°C in 5% CO_2_. The cells were passed every 4 to 5 days by trypsinization and were seeded into Corning flasks at 1.2×10^6^ cells/flask, resulting in complete confluent (≈2×10^6^ cells/flask) cultures in 4 days. For ARPE-19 monolayer culture, they were seeded at 2×10^5^ cells/cm^2^ onto Transwell filters (Costar, 12 mm diameter, 0.4ììm pore size), which had been coated with 160 µl of a 1∶30 dilution of Matrigel (BD Biosciences, Bedford, MA) in DMEM/F12 and air-dried overnight. DMEM/F12 medium supplemented with 1% FBS was used to culture these cells and was changed every 4 days.

### Western blot

ARPE-19 cells and PBMCs were rinsed with ice-cold PBS and cells were lysed with lysis buffer containing 50 mM Tris-HCl (pH 7.4), 150 mM NaCl, 1% Triton X-100, 1% sodium deoxycholate, 0.1% SDS, 2 mM EDTA, 100 µM phenylmethylsulfonylfluoride. The cell lysate was centrifuged and supernatant was collected. Protein concentration was determined with a protein assay (Bio-Rad, Richmond, CA). Laemmli gel loading buffer was added to the lysate and boiled for 7 minutes, after which proteins were separated on an SDS- polyacrylamide gel. Proteins were transferred to polyvinylidene difluoride membranes (Millipore, Bedford, MA), blocked by 5% skim milk at 37°C for 2 hours, and incubated with the primary antibodies anti-IL-17RA, anti-IL-17RC (Santa Cruz, CA) at 4°C for 16 hours, followed by a horseradish peroxidase-conjugated secondary antibody at 37°C for 1 hour. The membranes were further developed using a chemiluminescent detection kit (Cell Signaling Technology, Beverly, MA).

### Enzyme-Linked Immunosorbent Assay (ELISA)

ARPE-19 cells were maintained in DMEM/F12 medium containing 10% FBS for 4 days to become confluent. Before stimulation, cells were serum-starved for 24 hours in medium with 1% FBS. ARPE-19 cells were not stimulated (controls) or stimulated with 10, 50 or 100 ng/ml recombinant IL-17A, IL-17F or IL-22 (R&D Systems, Minneapolis, MN) for 24 hours. The collected supernatants were centrifuged (1000 g X 5 minutes) to remove particulates and stored at −70°C until analysis. CXCL8, CCL2, CCL20, CXCL10 and IL-6 were measured using human ELISA development kits (Duoset; R&D Systems, Minneapolis, MN). Each stimulation experiment was repeated four times.

### Measurement of TER

TER was measured using a commercial electrical resistance system (Millicell; Millipore, Bedford, MA). All TER measurements were performed within 3 min after removal of filters from the incubator. Net TERs were calculated by subtracting the value of a blank, Matrigel-coated filter without cells from the experimental value. Final resistance-area products (Ω/cm^2^) were obtained by multiplying the net TERs by the effective growth area. Measurements were performed every 3 days within the first 16 days and every other day or every day during the following culture. After TER stabilization, the ARPE-19 monolayer was incubated with or without one of various stimulants: IL-17A(50 ng/ml), IL-17F(50 ng/ml) or IL-22(50 ng/ml). The medium with or without stimulants was changed every other day. Measurements were repeated at least four times for each filter, and each experiment was repeated at least four times using 4 filters.

### Permeability Assay

The paracellular permeability of the ARPE-19 monolayer was evaluated by measuring the passive permeation of FITC-dextran (4 kDa, Sigma-Aldrich, Shanghai, China) across confluent cells grown on filters. Three weeks later, the ARPE-19 monolayer was treated with IL-17A(50 ng/ml), IL-17F(50 ng/ml) or IL-22(50 ng/ml) for 6 days. The medium was changed every 2 days, and 1000 µg/ml FITC-dextran was added to the upper chamber on day 6 following stimulation. Samples(100 µl) were taken from the upper and lower chamber 24 hours after addition of FITC-dextran. The concentration of FITC-dextran in these samples was quantified by spectrophotometry. The diffusion rate was expressed as a percentage and calculated as follows: (amount of dextran lower chamber)×100/(amount of dextran upper chamber) [15]. Each experiment was repeated four times.

### Immunocytochemistry

The expression of IL-17RA, IL-17RC and IL-22R (R&D Systems, Minneapolis, MN) as well as the formation of tight junction proteins in the ARPE-19 monolayer was examined by immunocytochemistry. The staining of tight junction proteins including occludin and ZO-1 was performed using the ARPE-19 cells cultured under conditions identical to these in the permeability assay. ARPE-19 cells grown on coverslips or filters were fixed with 4% paraformaldehyde in phosphate-buffered saline (PBS) for 20 minutes at room temperature (RT) and rinsed three times. The ARPE-19 cells cultured on coverslips were directly used for the detection of IL-17RA, IL-17RC and IL-22R. The ARPE-19 monolayer on filters was permeabilized by 0.2% Triton X-100 in PBS for 20 minutes and used for the assay of tight junction proteins. For the immunocytochemistry staining, the ARPE-19 cells on coverslips or filters were incubated with 10% goat or rabbit serum at RT for 30 minutes to exclude nonspecific staining. These cells were then incubated with each of rabbit anti-occludin, ZO-1 (Zymed-Invitrogen Carlsbad, CA), IL-17RC antibodies, mouse anti-IL-22R or goat anti-IL-17RA antibodies overnight at 4°C. Rabbit IgG, Mouse IgG_1_ or Goat IgG were used as isotype controls. After washing with PBS for three times, the cells were incubated with secondary antibody–labeled fluorescent dye (Dylight 488, Dylight 594 or rhodamine; Jackson Immunoresearch Laboratories, West Grove, PA) for 30 minutes at RT and then with DAPI for staining of nuclei for 2 minutes. Photographs were taken by a laser confocal microscope (TCSSP2; Leica, Germany).

### Statistical Analysis

Student's t-test was applied using SPSS 12.0. A p<0.05 was considered significant for all experiments. Values were presented as means±SEM.

## References

[1] Harrington LE, Hatton RD, Mangan PR, Turner H, Murphy TL (2005). Interleukin 17-producing CD4+ effector T cells develop via a lineage distinct from the T helper type 1 and 2 lineages.. Nat Immunol.

[2] Wilson NJ, Boniface K, Chan JR, McKenzie BS, Blumenschein WM (2007). Development, cytokine profile and function of human interleukin 17-producing helper T cells.. Nat Immunol.

[3] Amadi-Obi A, Yu CR, Liu X, Mahdi RM, Clarke GL (2007). TH17 cells contribute to uveitis and scleritis and are expanded by IL-2 and inhibited by IL-27/STAT1.. Nat Med.

[4] Caspi R (2008). Autoimmunity in the immune privileged eye: pathogenic and regulatory T cells.. Immunol Res.

[5] Park H, Li Z, Yang XO, Chang SH, Nurieva R (2005). A distinct lineage of CD4 T cells regulates tissue inflammation by producing interleukin 17.. Nat Immunol.

[6] Yang XO, Chang SH, Park H, Nurieva R, Shah B (2008). Regulation of inflammatory responses by IL-17F.. J Exp Med.

[7] Li Z, Liu B, Maminishkis A, Mahesh SP, Yeh S (2008). Gene expression profiling in autoimmune noninfectious uveitis disease.. J Immunol.

[8] Harper EG, Guo C, Rizzo H, Lillis JV, Kurtz SE (2009). Th17 cytokines stimulate CCL20 expression in keratinocytes in vitro and in vivo: implications for psoriasis pathogenesis.. J Invest Dermatol.

[9] McAllister F, Henry A, Kreindler JL, Dubin PJ, Ulrich L (2005). Role of IL-17A, IL-17F, and the IL-17 receptor in regulating growth-related oncogene-alpha and granulocyte colony-stimulating factor in bronchial epithelium: implications for airway inflammation in cystic fibrosis.. J Immunol.

[10] Kuestner RE, Taft DW, Haran A, Brandt CS, Brender T (2007). Identification of the IL-17 receptor related molecule IL-17RC as the receptor for IL-17F.. J Immunol.

[11] Wolk K, Witte E, Witte K, Warszawska K, Sabat R (2010). Biology of interleukin-22.. Semin Immunopathol.

[12] Aggarwal S, Gurney AL (2002). IL-17: prototype member of an emerging cytokine family.. Journal of Leukocyte Biology.

[13] Hymowitz SG, Filvaroff EH, Yin JP, Lee J, Cai L (2001). IL-17s adopt a cystine knot fold: structure and activity of a novel cytokine, IL-17F, and implications for receptor binding.. EMBO J.

[14] Tohyama M, Hanakawa Y, Shirakata Y, Dai X, Yang L (2009). IL-17 and IL-22 mediate IL-20 subfamily cytokine production in cultured keratinocytes via increased IL-22 receptor expression.. Eur J Immunol.

[15] Kebir H, Kreymborg K, Ifergan I, Dodelet-Devillers A, Cayrol R (2007). Human TH17 lymphocytes promote blood-brain barrier disruption and central nervous system inflammation.. Nat Med.

[16] Chi W, Yang P, Li B, Wu C, Jin H (2007). IL-23 promotes CD4+ T cells to produce IL-17 in Vogt-Koyanagi-Harada disease.. J Allergy Clin Immunol.

[17] Chi W, Zhu X, Yang P, Liu X, Lin X (2008). Upregulated IL-23 and IL-17 in Behcet patients with active uveitis.. Invest Ophthalmol Vis Sci.

[18] Holtkamp GM, Kijlstra A, Peek R, de Vos AF (2001). Retinal pigment epithelium-immune system interactions: cytokine production and cytokine-induced changes.. Prog Retin Eye Res.

[19] Chang SH, Dong C (2009). IL-17F: regulation, signaling and function in inflammation.. Cytokine.

[20] Kerr EC, Copland DA, Dick AD, Nicholson LB (2008). The dynamics of leukocyte infiltration in experimental autoimmune uveoretinitis.. Prog Retin Eye Res.

[21] Yao Z, Fanslow WC, Seldin MF, Rousseau AM, Painter SL (1995). Herpesvirus Saimiri encodes a new cytokine, IL-17, which binds to a novel cytokine receptor.. Immunity.

[22] Leung KW, Barnstable CJ, Tombran-Tink J (2009). Bacterial endotoxin activates retinal pigment epithelial cells and induces their degeneration through IL-6 and IL-8 autocrine signaling.. Mol Immunol.

[23] Ke Y, Jiang G, Sun D, Kaplan HJ, Shao H (2009). Retinal Astrocytes respond to IL-17 differently than Retinal Pigment Epithelial cells.. J Leukoc Biol.

[24] Ishihara K, Hirano T (2002). IL-6 in autoimmune disease and chronic inflammatory proliferative disease.. Cytokine Growth Factor Rev.

[25] Hirota K, Yoshitomi H, Hashimoto M, Maeda S, Teradaira S (2007). Preferential recruitment of CCR6-expressing Th17 cells to inflamed joints via CCL20 in rheumatoid arthritis and its animal model.. J Exp Med.

[26] Reboldi A, Coisne C, Baumjohann D, Benvenuto F, Bottinelli D (2009). C-C chemokine receptor 6-regulated entry of TH-17 cells into the CNS through the choroid plexus is required for the initiation of EAE.. Nat Immunol.

[27] Zrioual S, Ecochard R, Tournadre A, Lenief V, Cazalis MA (2009). Genome-wide comparison between IL-17A- and IL-17F-induced effects in human rheumatoid arthritis synoviocytes.. J Immunol.

[28] Abe T, Sugano E, Saigo Y, Tamai M (2003). Interleukin-1beta and barrier function of retinal pigment epithelial cells (ARPE-19): aberrant expression of junctional complex molecules.. Invest Ophthalmol Vis Sci.

[29] Zech JC, Pouvreau I, Cotinet A, Goureau O, Le Varlet B (1998). Effect of cytokines and nitric oxide on tight junctions in cultured rat retinal pigment epithelium.. Invest Ophthalmol Vis Sci.

[30] Buzza MS, Netzel-Arnett S, Shea-Donohue T, Zhao A, Lin CY (2010). Membrane-anchored serine protease matriptase regulates epithelial barrier formation and permeability in the intestine.. Proc Natl Acad Sci U S A.

[31] Miura Y, Klettner A, Roider J (2010). VEGF-antagonists decrease barrier function of retinal pigment epithelium in vitro -possible participation of intracellular glutathione.. Invest Ophthalmol Vis Sci.

